# Do Children with SLI Use Verbs to Predict Arguments and Adjuncts: Evidence from Eye Movements During Listening

**DOI:** 10.3389/fpsyg.2015.01917

**Published:** 2016-01-06

**Authors:** Llorenç Andreu, Mònica Sanz-Torrent, Javier Rodríguez-Ferreiro

**Affiliations:** ^1^Grup de Recerca en Cognició i Llenguatge, Estudis de Psicologia i Ciències de l’Educació, Universitat Oberta de CatalunyaBarcelona, Spain; ^2^Grup de Recerca en Cognició i Llenguatge, Departament de Psicologia Bàsica, Universitat de BarcelonaBarcelona, Spain; ^3^Institut de Recerca en Cervell, Cognició i Conducta (IR3C), Universitat de BarcelonaBarcelona, Spain

**Keywords:** specific language impairment, language comprehension, argument structure, arguments, adjuncts, eye movements

## Abstract

Different psycholinguistic theories have suggested the importance of verb semantics in rapidly anticipating upcoming information during real-time sentence comprehension. To date, no study has examined if children use verbs to predict arguments and adjuncts in sentence comprehension using children with specific language impairment (SLI). Twenty-five children with SLI (aged 5 years and 3 months to 8 years and 2 months), 25 age-matched controls (aged 5 years and 3 months to 8 years and 2 months), 25 MLU-w controls (aged 3 years and 3 months to 7 years and 1 month), and 31 adults took part in the study. The eye movements of participants were monitored while they heard 24 sentences, such as *El hombre lee con atención un cuento en la cama* (translation: *The man carefully reads a storybook in bed*), in the presence of four depicted objects, one of which was the target (*storybook*), another, the competitor (*bed*), and another two, distracters (*wardrobe* and *grape*). The proportion of looks revealed that, when the meaning of the verb was retrieved, the upcoming argument and adjunct referents were rapidly anticipated. However, the proportion of looks at the theme, source/goal and instrument referents were significantly higher than the looks at the locatives. This pattern was found in adults as well as children with and without language impairment. The present results suggest that, in terms of sentence comprehension, the ability to understand verb information is not severely impaired in children with SLI.

## Introduction

Understanding sentences in real time requires the rapid activation of conceptual and linguistic information that combines bottom–up and top–down processes. These processes go beyond knowledge of grammar and single word identification, because this information has to be integrated with knowledge about the world in a concrete situation. So, language comprehension requires a complex relationship between language and thought, which have to be linked quickly due to the speed at which the input unfolds.

In a language comprehension process, verb semantics play an important role according to different psycholinguistic theories. Research in this field has shown how interpretation of linguistic input affects the course of comprehension, enabling the upcoming appropriate referent to be anticipated rapidly (e.g., Altmann and Kamide, 1999; Sedivy et al., 1999; Chambers et al., 2002; Hanna et al., 2003; Kamide et al., 2003a; Runner et al., 2003; Knoeferle et al., 2005). The activation of a verb involves various types of information (argument structure, thematic roles, subcategorization restrictions, selectional restrictions, etc.), which are activated in real time when a verb is retrieved in the process of language comprehension.

Argument structure is a construct within linguistic theory that specifies the relationship between the semantics of a lexical item and its syntactic expression, and, as such, serves as an important interface between lexis, syntax and semantics (e.g., Levin and Rappaport Hovav, 1996; Jackendoff, 2002; Grimshaw, 2005). Carnie (2013) showed that the argument structure of a verb includes the number of arguments (one, two, or three) that a verb requires in a particular predicate (intransitive, transitive, or ditransitive). Arguments can play different thematic roles with respect to the predicate (agent, experiencer, source, goal, instrument, location, etc.). Moreover, a verb imposes restrictions on the categories of its complements (subcategorization restrictions) as well as semantic restrictions on the features that the arguments of the verb must comply with (selectional restrictions).

Some psycholinguistic parsing theories (Frazier and Clifton, 1996; Boland and Boehm-Jernigan, 1998; Stevenson, 1998) and formal theories of syntax, including principle and parameter approaches (Chomsky, 1981), lexical-functional grammar (Kaplan and Bresnan, 1982) and role and reference grammar (Van Valin and Lapolla, 1997), theoretically distinguished between arguments and adjuncts. This distinction has also been theoretically important in psycholinguistics, especially in cases where parsing theories must explain how syntactic representations are built incrementally during sentence comprehension. In general, arguments are considered as the essential participants that must be specified lexically for a sentence to have full meaning. Arguments have the thematic role of agent, theme, source, goal, instrument, etc. However, adjuncts are structurally dispensable information whose expression is optional, such as locatives or comitatives. For example, in the sentence *Aina goes to the swimming pool with her red towel*, *the swimming pool* express the thematic role of the goal (argument) and *the red towel*, the comitative (adjunct), but in *Aina buys a red towel at the swimming pool*, *the red towel* describes the theme (argument) and the swimming pool, the locative (adjunct). Thus, adjuncts are viewed as supplements that are not selected by the verb but can complete its meaning, and their deletion does not cause ungrammaticality. In general, instruments, locatives and comitatives, among others, are considered adjuncts (Bosque and Demonte, 1999).

In this vein, there is one language disorder that is characterized by developmental delays in verbal abilities that can affect both expressive and receptive language, namely specific language impairment, or SLI (Bishop, 1997; Leonard, 1998). SLI is a developmental language disorder in the absence of clear neurological, sensory-motor, non-verbal cognitive or social-emotional deficits. Verbs have been proposed as an area of particular difficulty for children with SLI (Bishop, 1997; Conti-Ramsden and Jones, 1997; Verhoeven and Van Balkom, 2004). They show a substantial delay in the use and understanding of verbs and functional morphology. The speech of SLI children is characterized by having greater than average misuse and the dropping of inflectional morphology (*-s, -ed*) and closed class function words (*the, a*, etc.) (Leonard, 1995; Rice et al., 1995; Rice and Wexler, 1996, 1997; Leonard et al., 1997).

Other studies suggest that children with SLI may also have particular problems with verb semantics and particularly with argument structure (e.g., Fletcher, 1991; Roberts et al., 1994; Grela and Leonard, 1997; Thordardottir and Weismer, 2002; Grela, 2003; Pizzioli and Schelstraete, 2008; Sanz-Torrent et al., 2011; Andreu et al., 2012). Most of these studies were carried out in English and their results are contradictory in some cases. While some studies find that children with SLI make omissions and errors in the production of arguments similar to those of age controls (Rice and Bode, 1993; Lee, unpublished), other studies have suggested that children with SLI have particular problems with verb argument structure. These studies have shown that children with SLI use significantly fewer argument types (i.e., thematic roles), argument structure types (i.e., verbs with a different argument structure: intransitive, transitive, and ditransitive verbs) and verb alternations than age-matched children (Thordardottir and Weismer, 2002); they omit more obligatory arguments than age-matched controls (Fletcher, 1991; Roberts et al., 1993; Grela and Leonard, 1997; Grela, 2003; Sanz-Torrent et al., 2011; Andreu et al., 2012) and make errors in a much wider variety of verbs than MLU controls (King and Fletcher, 1993). Moreover, de Jong (1999) showed that children with SLI produced fewer verbs and that the complements they used were not very diverse or complex.

Only a small number of studies have analyzed SLI children’s use of adjuncts. Fletcher and Garman (1988) examined the use of adjuncts expressing time, location or manner of action in the spontaneous speech of normally developing children and those with SLI. The SLI group used temporal adverbials less frequently than normally developing children when the context did not provide cues that specified reference time. Johnston and Kamhi (1984) found adverbials to be used less frequently by a group of children with SLI than by a group of MLU controls. These findings do not clearly point to a single source of verb-related problems. Some of the problems could be due to strictly lexical limitations such as incomplete information in the verb’s lema or lexical concept, but the adjunct difficulties suggest that limitations could not have been caused by the verb.

Although there is a general consensus on the linguistic profile of those with SLI, there is considerable debate regarding difficulties with verb semantics in children with SLI. Broadly speaking, two classes of explanations exist in current literature. On the one hand, some investigations attribute these difficulties to deficits or immaturities in semantic representations (e.g., Thordardottir and Weismer, 2002; Sanz-Torrent et al., 2011; Andreu et al., 2012). This interpretation is based on the idea that the degree of knowledge represented in children’s semantic verb lexicon causes children with SLI to exhibit more argument omissions and fewer thematic roles, argument structure types and verb alternatives than their peers (e.g., Thordardottir and Weismer, 2002; Grela, 2003; Sanz-Torrent et al., 2011; Andreu et al., 2012). Andreu et al. (2012) investigated the picture naming of nouns and verbs with different argument structures in children with SLI. They compared the response times and naming accuracy for nouns and verbs with differing argument structures among Spanish-speaking children with and without language impairment and adults. The results showed that all groups produced more correct responses and were faster with nouns than all the verbs together. In regard to verb-type accuracy, there were no differences between groups in naming one-argument verbs. However, for both two- and three-argument verbs, children with SLI were less accurate than adults and age-matched controls, but similar to the MLU-matched controls. For verb-type latency, children with SLI were slower than both the age-matched controls and adults for one- and two-argument verbs, while no differences were found in three-argument verbs. No differences were found between children with SLI and the MLU-matched controls for any verb type. They concluded that children with SLI may have problems encoding verb semantic representations. Sanz-Torrent et al. (2011) studied the verb production and argument structure of Catalan and Spanish children with SLI using different methodologies. The first was an observational study which used a spontaneous-talk longitudinal sample. The second was an experimental sentence-naming task based on event video observation. The third comprised an experimental sentence-naming task with static images that differed in verb argument complexity. Although the specific data varied according to the methodology used, results showed that children with SLI had particular difficulty in producing verbs with a highly complex argument structure, often omitting obligatory arguments. It was concluded that both processing limitations and deficits in the semantic representation of verbs could play a role in these difficulties. Thordardottir and Weismer (2002) analyzed speech samples from 50 children with SLI. They found that children with SLI used significantly fewer argument types, argument structure types, and verb alternations than age-matched children with normal language (NL). They suggested that these differences were not merely attributable to production limitations such as utterance length but that they could also be due to an incomplete argument structure representation for verbs.

In contrast, other accounts attribute these difficulties to processing limitations (Leonard, 1998; Weismer et al., 1999; Montgomery, 2000; Miller et al., 2001). In support of this, several studies have emphasized that children with SLI take longer to complete a certain amount of work in a given unit of time. Consequently, the greater the linguistic complexity of the sentences, the greater the difficulty exhibited by children with SLI. From this perspective, Grela (2003) analyzed the omission of subject arguments in English-speaking children with SLI. Participants were asked to produce sentences of varied argument structure complexity using a story completion task. The results indicated that both children with SLI and MLU controls omitted more grammatical subject arguments in ditransitive sentences than in sentences with intransitive and transitive verbs. In addition, more children with SLI omitted subjects as the linguistic complexity of the sentence increased. This effect was not found in the control children, who never omitted subjects, regardless of the increase in argument structure complexity. Grela argued that these results supported the notion that grammatical errors in both children with SLI and their younger, normal counterparts could be due to problems in processing complex linguistic information, rather than limitations in linguistic knowledge. Pizzioli and Schelstraete (2008) studied the effect of argument structure complexity in French children with SLI. They showed that more complex argument structures elicited the highest number of grammatical morpheme omissions and that this effect was independent of sentence length. The authors suggested that this data supported the hypothesis that grammatical morpheme deficit in children with SLI depended at least in part on limited processing capacities.

Most of the previous studies focused on language production and were based on off-line methodologies. In contrast, the present work is a language comprehension study based on the “visual world paradigm” (Cooper, 1974; Tanenhaus et al., 1995), which uses eye movements to track the detailed incremental nature of spoken language processing in real time. Studies carried out by Altmann and Kamide (1999), Kamide et al. (2003a,b), Boland (2005), Knoeferle et al. (2005), and Knoeferle and Crocker (2006, 2007) have shown that verb semantics were used to determine the subsequent referents during real-time sentence comprehension. In all of these studies, listeners showed anticipatory eye movements that reflected real-time activation of verb meaning. Boland (2005) showed that visual world studies are well suited to the investigation of how the recognition of a spoken verb allows new entities to be introduced into the discourse via their argument structure, the associated thematic roles and the selectional restrictions. Boland (2005, Experiment 3) showed that there were more anticipatory eye movements to potential arguments than adjuncts, beginning 500ms after the onset of the verb.

In regard to children, only a handful of eye-tracking studies have explored the use of verb information to achieve anticipatory processing. Nation et al. (2003) conducted a study similar to Kamide et al. (2003a). Skilled and less-skilled child comprehenders were presented four images (the target, for example, *cake*, and three distracters) while listening to sentences in which all objects satisfied the semantic requirements of the verb (e.g., *Jane watched her mother choose the cake*) or only one of them did (e.g., *Jane watched her mother eat the cake*). Upon hearing *eat*, all the children made faster anticipatory eye movements to the target object than when hearing *move*. Less-skilled comprehenders did not differ from the controls in the speed of their anticipatory eye movements. However, they made more and shorter fixations on target objects than the skilled comprehenders. Brock et al. (2008) studied the use of verb information during spoken sentences in adolescents with autism spectrum disorder and in language-matched peers. In the target-present condition, each display comprised the target object (e.g., *hamster*), a phonological competitor (e.g., *hammer*) and two unrelated distracters. In the target-absent condition, each display contained a phonological competitor of the target word and three unrelated distracters. Results showed that the eye movement of the two groups was similarly affected by context. Moreover, across both groups, the effect of sentence context was reduced in individuals with relatively poor language skills. Finally, more recently, Andreu et al. (2013) conducted three spoken language comprehension eye-tracking experiments with Spanish-speaking adults and children with and without SLI. Participants listened to sentences like *The boy carefully trims the paper* in the presence of four depicted objects (the target, *paper*, and three distracters). Results revealed that children with SLI were able to recognize and retrieve the meaning of the verb quickly enough to anticipate the upcoming semantically appropriate referent (Experiment 1). Experiments 2 and 3 revealed that, for all the groups of children, the anticipatory eye movements were also modulated by the semantic fit/typicality of the object serving as the patient/theme of the verb. Children with SLI did differ from age-matched controls, but only slightly in terms of overall anticipatory looks at the target object. In addition, no differences were found between children with SLI and the control children matched for mean length of utterance (MLU).

As we have seen, several studies have shown that children with SLI present difficulties in producing arguments and adjuncts. Most of them are language production studies based on off-line methodologies. Moreover, eye-tracking studies have provided evidence that adults use verb semantics to restrict proactively the domain of subsequent reference (e.g., Altmann and Kamide, 1999; Kamide et al., 2003a; Boland, 2005; Knoeferle et al., 2005; Knoeferle and Crocker, 2006, 2007). In this paper, we explore in some detail the verb-based anticipatory comprehension skills of children with SLI. Concretely, we analyze if these children are able to access the meaning of verbs, embedded in a sentence, rapidly enough so as to anticipate their possible arguments and adjuncts.

Although accounts on processing limitations predict that children with SLI may have problems with complex and long sentences, only simple and canonical word order sentences were used in the present work. The goal was to analyze the alternative hypothesis in greater detail. In a previous study, Andreu et al. (2013) found that children with SLI were able to anticipate argument referents in simple sentences (*the boy trims carefully the paper*) and only differed from age-matched controls in the overall number of anticipatory looks at the target object, with no differences being found with the MLU group. This work suggested that these differences arose because children with SLI have slightly smaller verb lexicons or are less certain of the semantics of some verbs than age-matched controls. In our study, we selected arguments and adjuncts. Arguments are essential participants that must be specified in the sentence, but the expression of adjuncts is optional. Previous studies in language production have shown that children with SLI have more omissions of obligatory arguments and adjuncts than age-matched controls (e.g., Fletcher, 1991; Roberts et al., 1993; Grela and Leonard, 1997; Thordardottir and Weismer, 2002; Grela, 2003; Pizzioli and Schelstraete, 2008; Sanz-Torrent et al., 2011; Andreu et al., 2012) because they have deficits in the semantic representations of verbs. So, in this study, if, on the one hand, children with SLI do indeed have poor verb semantic representations, then fewer differences can be expected between the anticipatory looks for the arguments and adjuncts. Poorer verb semantic representation would mean that children with SLI would not expect that the essential participants in the predicate (arguments) would have to be specified in the sentence and their anticipation would be similar to the adjuncts. Moreover, it can be expected that children with SLI will be less accurate in anticipating both arguments and adjuncts with respect to their age-matched controls. In addition, under this account, SLI children’s performance will be more like that of linguistically-matched control children (i.e., MLU-matched) than of age-matched controls. On the other hand, if children with SLI do not have poor verb semantic representations, significant differences can be expected between the anticipatory looks to argument and adjuncts referents and no differences with respect to their age-matched controls.

## Materials and Methods

### Participants

All participants were native Spanish speakers and had normal or corrected-to-normal vision. Four groups took part in this study. The first consisted of 25 children (18 boys, 7 girls) with SLI, with ages ranging from 5 years and 3 months to 8 years and 2 months of age. The second group consisted of 25 children matched in terms of age, sex, and mother tongue with the children with SLI (18 boys, 7 girls), ranging 5 years and 3 months to 8 years and 2 months of age. The third group consisted of 25 children (18 boys and 7 girls) matched based on mean length of utterance by words (MLU-w), sex and mother tongue with the children with SLI (18 boys and 7 girls), ranging from 3 years and 3 months to 7 years and 1 month of age. MLU-w was obtained from speech samples taken from situations involving adult interaction. The recording of all the groups lasted 10 min. Samples were transcribed using the CHAT format (MacWhinney, 2000). The computation of MLU-w was carried out by calculating the MLU omitting imitations, repetitions, stereotypes, communicative routines and minor productions (that is, social words and others such as adverbs of affirmation or negation). Finally, the fourth group consisted of 31 adults (16 females and 15 males). They were students or junior faculty members at various universities in the Barcelona area with ages ranging from 18 to 42 years.

The children with SLI were selected according to standard criteria for diagnosing SLI (Stark and Tallal, 1981; Watkins, 1994; Leonard, 1998). Specifically, the children with SLI were tested to assess their non-verbal intelligence and level of language development. Tests included the Wechsler Intelligence Scale for Children (WISC-R, Spanish version; Wechsler et al., 1993) or the Kaufman Brief Intelligence Test (KBIT, Spanish version; Kaufman and Kaufman, 2004). Every child with SLI obtained a non-verbal IQ standard score of over 85. Language ability was assessed by language profiles following the Spanish protocol for the evaluation of language delay (AREL; Pérez and Serra, 1998), the Spanish version of Peabody Picture Vocabulary Test III (PPVT-III; Dunn et al., 2006) and the child language scale (Evaluación del Lenguaje Infantil, ELI; Saborit and Julián, 2003). The ELI test includes several subtests for lexical reception, lexical production, phonetics and pragmatics. Children with SLI had scores of at least a -1.25 standard deviation below average, both in Peabody III and ELI. Language profiles based on transcripts of spontaneous speech provided information about the children’s morpho-syntactic abilities in language production, from which it was determined that they showed a delay of at least 1 year (see Bishop, 1997). Children with SLI made very frequent use of non-finite forms, particularly the infinitive, far more than the age and MLU control groups. Moreover, children with SLI made more omissions in functional words such as pronouns and prepositions than their age-matched controls. We also calculated the MLU value in words for each child. Each child passed a hearing screening for each ear (25 dB at 500, 1000, 2000, and 4000 Hz.). With respect to neurological dysfunctions, the case histories of all the children were seen by an educational psychologist, to rule out any evidence of cerebral palsy or brain damage. With respect to oral structure and motor function, speech therapists examined the children to assess the shape, size and motor function of the speech organs, both active (tongue, lips, and jaw) and passive (buccal cavity, palate, and teeth), as well as respiratory dynamics, exhalation and rhythm. Motor function was assessed according to a protocol that used different practical exercises to verify that mobility was normal. With respect to physical and social interactions, educational psychologists drew up a report containing information about each child’s family background and aspects of their personality, such as self-esteem, sense of self-confidence and confidence in others, level of socialization, social abilities, degree of anxiety, etc. This information was used to verify that each child had no symptoms of impaired reciprocal social interaction or any restriction of activities. In addition, all the children selected for the study had been diagnosed with SLI by a speech-language therapist of school educational psychology services following the exclusion criteria established by Leonard (1998, p. 10) and were receiving language intervention.

The age-matched control group was equivalent in age (same year and ±2 months) and had the same mother tongue (Spanish) as their counterparts in the SLI group. Children were not selected if they had a history of speech therapy or psychological therapy. Moreover, teachers were asked to select children with NL development and academic performance for their age. All of the children selected came from state schools in Catalonia.

The MLU-w control group was equivalent in terms of linguistic level. Each child in the study group was paired with another child according to the MLU calculated in words (±0.6 words), sex and mother tongue. In addition, non-verbal intelligence and language ability was assessed in all the children selected in both the age and MLU control groups as being the same as those of the children in the SLI group. The parents of the children and adult participants gave written informed consent for participation in this study. A summary of descriptive data for the three groups of children is presented in **Table 1**.

**Table 1:** Age group, cognitive measurements, and language performance.

	Group		
	SLI group	Age controls	MLU-w controls
	Mean (*SD*)	Mean (*SD*)	Mean (*SD*)
Age (years)	6.69 (0.90)	6.72 (0.92)	5.51 (1.05)
NVIQ	96.1 (7.9)	106.3 (6.0)	93.13 (9.32)
PPVT-III	89.58 (9.56)	112.07 (14.37)	92 (12.87)
ELI-Phonetics^∗^	6.37 (4.27)	2.12 (2.23)	4.47 (3.87)
ELI-Receptive vocabulary^∗^	36.27 (18.84)	73.07 (17.97)	67.85 (26.13)
ELI-Expressive vocabulary^∗^	8.62 (1.8)	60.38 (15.06)	52.27 (28.84)
ELI-Pragmatics^∗^	53.64 (25.99)	80.38 (15.60)	62.56 (14.34)
**Morpho-syntax measures**			
MLU-w	3.95 (1.39)	6.86 (1.76)	3.97 (1.45)
Infinitives	0.18 (0.6)	0.07 (0.04)	0.06 (0.4)
Gerunds	0.01 (0.01)	0.006 (0.008)	0.01 (0.01)
Participles	0.008 (0.006)	0.002 (0.001)	0.001 (0.001)
Verb morphology errors	0.06 (0.05)	0.01 (0.01)	0.01 (0.01)
Function word error/omission	0.06 (0.05)	0.015 (0.013)	0.04 (0.03)

Chronological age in years; NVIQ, Non-verbal Intelligence Quotient in standard score (mean = 100; SD: 15); PPVT-III, Peabody Picture Vocabulary Test III, Spanish version) in standard score (mean = 100; SD: 15); ELI, Evaluación del Lenguaje Infantil); ELI-Phonetics in mean of number of errors; ELI-Receptive vocabulary, ELI-Expressive vocabulary and ELI-Pragmatics in percentiles; MLU-w (mean length of utterance by words). Morphosyntax measures in proportions. *Values only calculated with children under 6 years of age.

### Ethics Statement

The study was approved by the Ethics Committee of the Universitat Oberta de Catalunya. Adult participants and the parents of child participants gave their written informed consent for participation in this study.

### Stimuli

We selected different sentences in which arguments (themes, sources/goals, instruments) and adjuncts (comitatives and locatives) played different thematic roles with respect to the predicate. We categorized the sentences according to four conditions. Unlike Boland’s (2005) study which used complex sentences (i.e., sentences containing dependent clauses), we only used very simple sentences (i.e., sentences containing one independent clause) for the experiment with children. Moreover, we did not use any thematic role that involved people (i.e., agents, patients, recipients, etc.) because several studies have shown that images containing animate entities attract looks both in adults (e.g., Buswell, 1935; Yarbus, 1967; Friedman, 1979; Castelhano et al., 2008) and children (e.g., Andreu et al., 2012).

All the target arguments and adjuncts were placed immediately after the verb to avoid the effect of selectional restrictions that an intermediate argument could have. Moreover, we selected other possible verb arguments or adjuncts as competitors. As Spanish is a very flexible language that allows the canonical positions of the verb’s arguments to be changed, they competed for the upcoming argument in the sentence. These are the four conditions used in the study:

-Transitive verb/Theme: *La niña come despacio la tarta con la cuchara* (Pictures: Target: *tarta*; Competitor: *cuchara*; Distracters: *sombrero*, *dinosaurio*). [*The girl slowly eats the cake with the* spoon. Pictures: T: *cake*; C: *spoon*, D: *hat, dinosaur*].

In this condition, the verb requires a theme (argument) and the natural position in Spanish for the theme is after the verb. We put two adjuncts in the sentence: a locative or an instrument.

-Verb of motion/Source-Goal: *El hombre entra despacio en casa con la maleta* (Pictures: Target: *casa*; Competitor: *maleta*; Distracter: *luna*, *tractor*). [*The man slowly enters the house with the suitcase*. Pictures: T: *house*; C: *suitcase*; D: *moon, tractor*].

Verbs of motion require a location after the verb to express the source of a goal (arguments) of the event. In this condition, we also included a locative or comitative (adjuncts) as a competitor.

-Verb of action/Instrument: *La mujer esquia deprisa con el trineo por la montaña* (Pictures: Target: *trineo*; Competitor: *montaña*; Distractor: *vaso*, *playa*). [*The woman skis down the mountain fast with the sled.* Pictures: T: *sled*; C: *mountain*; D: *cup, beach*].

In the verb of action condition, the verb requires an instrument (argument). We did not select verbs of action or instruments that had the same root (*serrar-sierra* [to saw-saw]). Moreover, we introduced a locative (adjunct).

-Intransitive verb/Locative: *La niña duerme siempre en la cama con el osito* (Pictures: Target: *cama*; Competitor: *osito*; Distracter: *arbol, bombilla*). [*The girl always sleeps in bed with a teddy bear.* Pictures: T: *bed*; C: *teddy bear*; D: *tree, bulb*].

In this condition, we selected intransitive verbs and locatives (adjuncts) that had strong semantic relationships amongst each other. These locatives were then typical locatives for the verb. Moreover, we chose instruments and comitatives as competitors.

Twenty-four simple sentences were constructed. All contained the same structure: Noun Phrase (NP) + Verb + Adverb + Target Phrase (NP or Prepositional Phrase, PP) + PP, which always corresponded to Agent + Verb + Adverb + Theme/Instrument/Locative/Source/Goal + Instrument/Locative/Comitative. The distribution of the 24 target phrases was as follows: six themes, six instruments, six locatives, and six locations that had the thematic roles of goal (four of them) and source (two of them). In order to minimize the restrictive effect the verb has on subsequent elements, all the sentences began with one of four possible agents: *the woman*, *the man*, *the girl*, or *the boy*. These were randomly assigned to sentences and for every condition there were three male and three female. Twenty-four different verbs were used. An adverb or adverbial phrase was placed after the verb to establish a temporary space for processing verb information. Adverbs used denoted the manner of the action (*attentively*, *quickly, slowly, suddenly*, *carefully, sadly*, and *cheerfully*) or the temporal properties of the action (*always* and *every day*). The target phrases followed three locatives and three instruments in the “theme” condition, six comitatives in the “source/goal” condition, six locatives in the “instrument” condition and three comitatives and three instruments in the “locative” condition. The experimental sentences are given in the Appendix.

**FIGURE 1:**
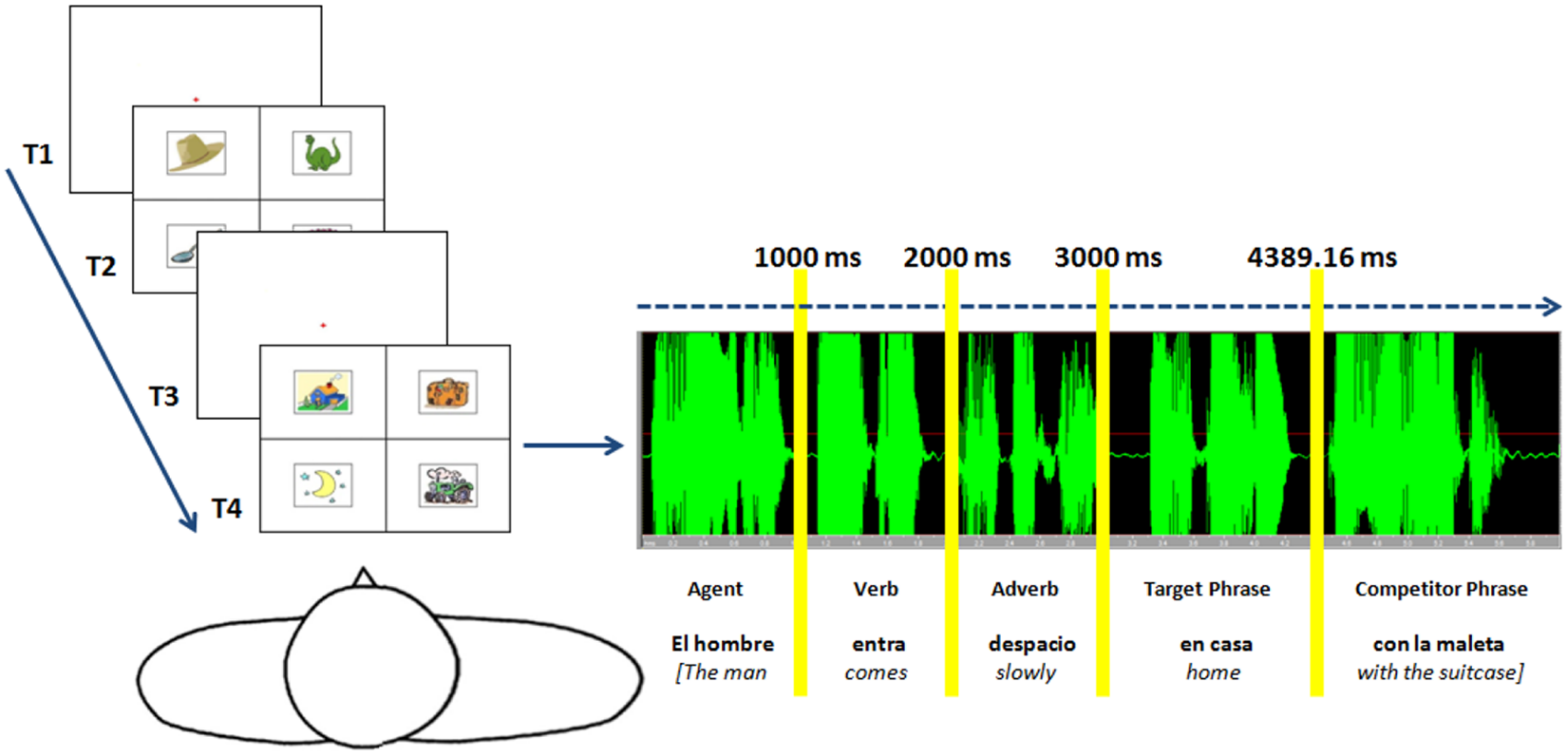
**Timeline of the visual stimuli and auditory sentences’ presentation.** The onset of the spoken sentence coincided with the onset of visual stimuli.

Sentences were recorded by a male native Spanish speaker at normal speech rate and sampled at 44,100 Hz. A digital audio editor was used to adjust each sentence so that the agent NP, the verb and the adverb each had a duration of one second (words + silence was 1000 ms). After the adverb, the target phrase started and had an average duration of 1389.16 ms when the competitor PP began (see **Figure 1**). Utterances sounded natural and unedited to adult native speakers. This facilitated subsequent analysis of data without having any effect on auditory stimuli (see Andreu et al., 2013).

Visual images were constructed and paired with each sentence. Each image consisted of four pictures located in the center of four quadrants on the screen. The background was white and two black lines (one vertical, one horizontal) were used to divide the four quadrants. The pictures were clip art images, which were at times altered using a professional image editing software package. The complexity of visual stimuli was assessed with digitized image compression (see Bates et al., 2003; Tuch et al., 2009). All images were compressed in JPEG format and file sizes were analyzed with a*t*-test that did not show significant differences between each target type: Themes vs. Source/Goals [*t*(5) = -0.74, *p* = 0.493], Themes vs. Instruments [*t*(5) = -1.58, *p* = 0.175], Themes vs. Locatives [*t*(5) = 0.42, *p* = 695], Source/Goals vs. Instruments [*t*(5) = -1.00, *p* = 0.363], Source/Goals vs. Locatives [*t*(5) = 1.66, *p* = 157] and Instruments vs. Locatives [*t*(5) = 2.26, *p* = 073]. In every image, there was one target picture depicting the target phrase, one that represented the following PP in the sentence (competitor) and two distracter pictures (see **Figure 1**). The distracters were pictures representing entities in the same category (locatives, instruments…) as the target and the competitor, but which were semantically impossible in the sentence. The position of the target, competitor and distracter picture in each quadrant was randomized. The audio and visual image for each item was merged together in a video file lasting 6000 ms, using VirtualDubMod software. In each video, the onset of the spoken sentence coincided with the onset of the visual stimuli. **Figure 1** shows an illustration of the timeline of the visual stimuli presentation and the auditory sentences.

### Procedure

Participants were seated at a distance of approximately 22″ in front of a *Tobii T120* eye tracker with an integrated 17″ TFT monitor. *Tobii Studio* software was used to present the stimuli and collect the eye-tracking data. Stimuli videos were made up of 800 × 600 pixel images presented on a screen set to 1024 × 768 pixels. The visual angle of each object subtended approximately 13°, well above the 0.5-degree accuracy of the eye tracker. When placed 57 cm from the participants, the four quadrants on the screen had an eccentricity of 5.1° and covered an approximate area of 4.2° × 4.7°. The stimuli sounds were presented to participants via a mono channel split to two loudspeakers positioned on either side of the viewing monitor. Eye position was sampled at 120 Hz (∼8 ms intervals).

A nine-point calibration was carried out at the beginning of the experiment. The *Tobii Studio* software automatically validated calibrations and the experimenter could, if required, repeat the calibration process if validation was poor. Calibration took approximately 20 s. Participants were instructed to listen to the sentences, inspect the images, and try to understand both sentences and depicted scenes. There were four practice trials before the experimental task (one per condition) to acquaint the participant with the flow of events. The test videos were presented in random order in two blocks containing 12 sentences each (three of each condition). All the participants were given both blocks. Between each trial, participants were first presented with a crosshair (on which they had been instructed how to fixate) for approximately 2000 ms so that their direction of gaze in each trial would start from the same point (the center of the screen that corresponded with the intersection of the two lines that divided the four quadrants).

### Analysis

In “visual world paradigm” experiments (Tanenhaus et al., 1995), participants view a scene and listen to speech containing references to objects in the scene. Changes over time in the distribution of looks at elements in the scene are taken as an index of underlying linguistic processing. In this study, we wish to determine whether verb-thematic information constrains referential searches. Therefore, the proportion of looks from verb onset to target phrase onset is the best index to evaluate moment-by-moment if verb information becomes quickly available and if it is used to derive expectations about upcoming referents.

For this purpose, eye position data obtained from the *Tobii Studio* software were used to calculate the proportion of looks made at the target picture by participants (as defined by the rectangles) before the name had been mentioned. To calculate looks toward the target, a value of one was given to every eye-tracking sample that fell within the target region; otherwise it was given a zero. We calculated the proportion of looks sample by sample by using the mean of looks for each image and each participant. We then compared whether anticipatory eye movements toward the target pictures differed depending on the type of the thematic roles. As in Trueswell et al. (1999), we rejected trials in which more than 33% of the frames presented track loss. Track loss is the non-registering of eye position data. Track loss occurs when the eye-tracking software fails to properly detect the pupil. This may occur as a result of the eyelids and/or eyelashes occluding portions of the pupil (e.g., when the subject blinks). After exclusion of these trials, participants who did not have at least 50% of the trials for each condition were removed. The mean percentage of track loss in adults was 4.92%, resulting in the need to drop eleven trials. The age control group had 7.62% track loss, and 32 trials were dropped. The MLU control group had 7.20% track loss, and 30 trials were dropped. The SLI group had 10.45% track loss and 33 stimuli and three participants were dropped.

## Results

**FIGURE 2:**
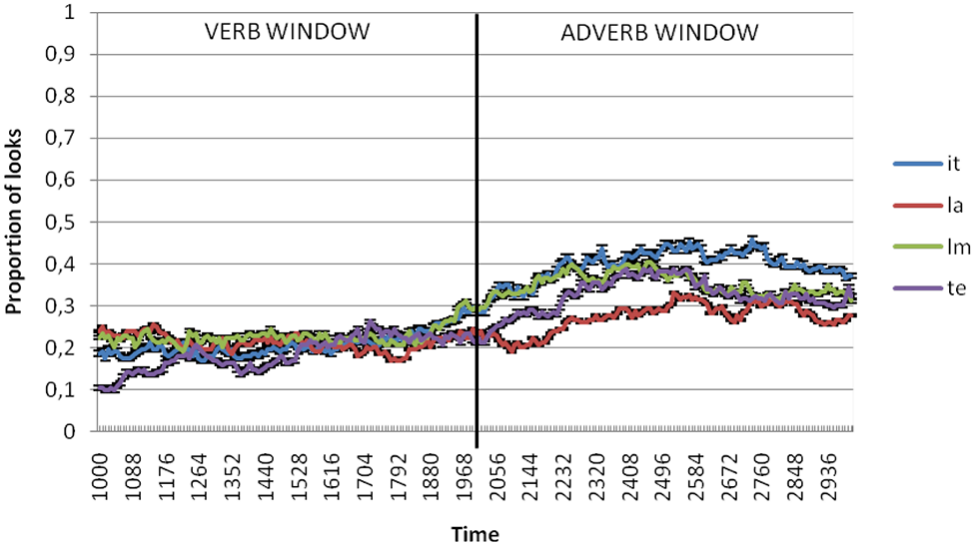
**Proportion of looks at theme (te), instrument (it), locative (la) and source/goal (lm) referents in verb and adverb windows in adults**.

**Figures 2** through **5** present the proportion of looks at the referent targets over time for adult, control Age, MLU and SLI groups. The black vertical line in the graphs divides the two temporal windows selected for analysis of the anticipatory eye movements at the target. The first was from the verb onset to offset (that included 1000–2000 ms from video onset) and the second between the adverb onset and offset (that included 2000–3000 ms from video onset). Because these means were proportions, they were first transformed using the Empirical Logit transformation (see Barr, 2008). By participants and by items analyses of variance (ANOVAs) were conducted over the means of the proportional data for each window of analysis with group (SLI, control age, MLU, adults) and argument type (Theme, Source/Goal, Instrument, Locative) as independent variables.

**FIGURE 3:**
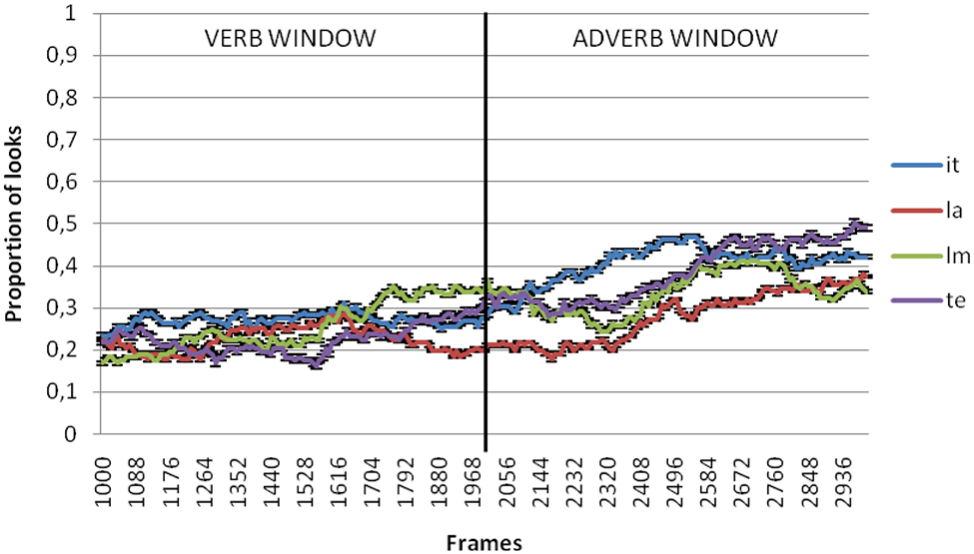
**Proportion of looks at theme (te), instrument (it), locative (la) and source/goal (lm) referents in verb and adverb window in age control group**.

**FIGURE 4:**
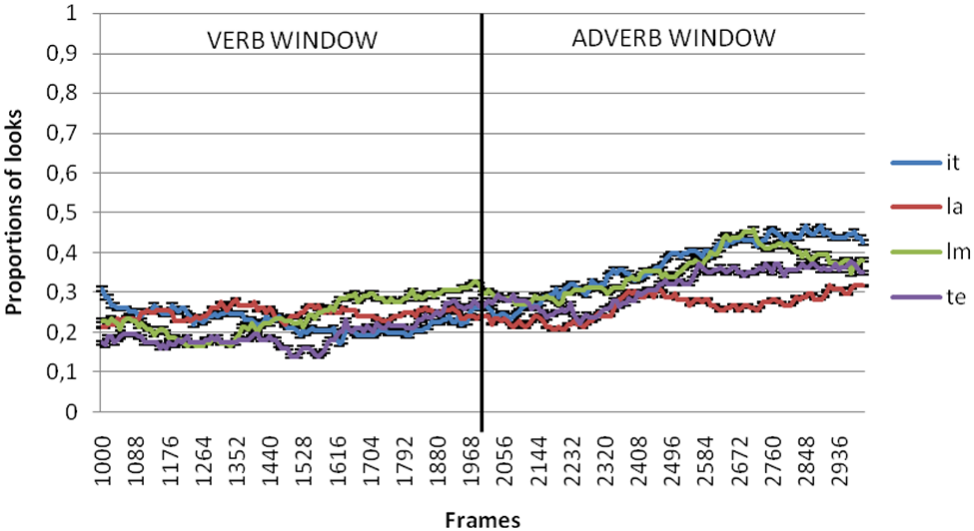
**Proportion of looks at theme (te), instrument (it), locative (la) and source/goal (lm) referents in verb and adverb window in MLU group**.

**FIGURE 5:**
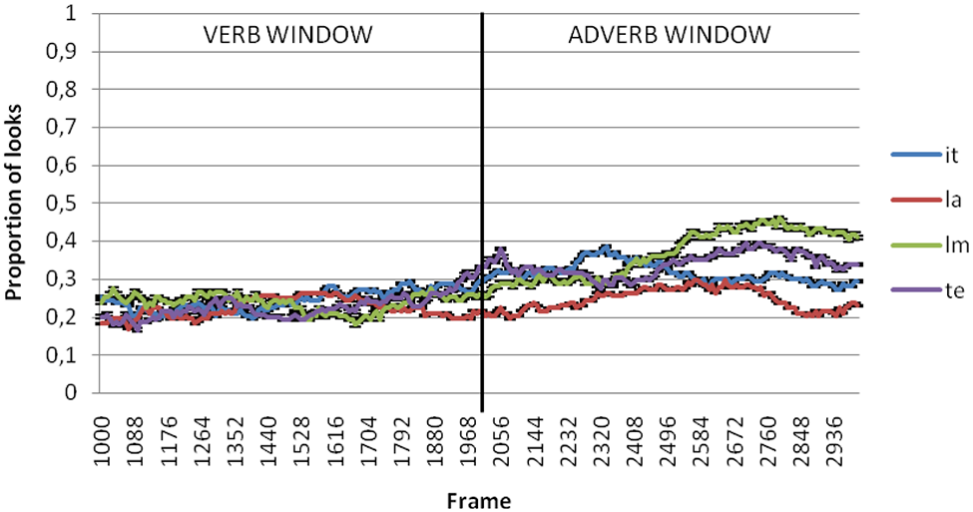
**Proportion of looks at theme (te), instrument (it), locative (la) and source/goal (lm) referents in verb and adverb window in SLI group**.

In the verb window (1000–2000 ms), results showed no significant effects of neither argument type [*F*_1_(3,306) = 2.497, *p* = 0.060; *F*_2_(3,80) = 0.746, *p* = 0.528] nor due to group [*F*_1_(3,102) = 0.147, *p* = 0.93; *F*_2_(3,80) = 0.072, *p* = 0.975] or the interaction between these two variables [*F*_1_(9,306) = 0.502, *p* = 0.873; *F*_2_(9,80) = 0.167, *p* = 0.997].

**FIGURE 6:**
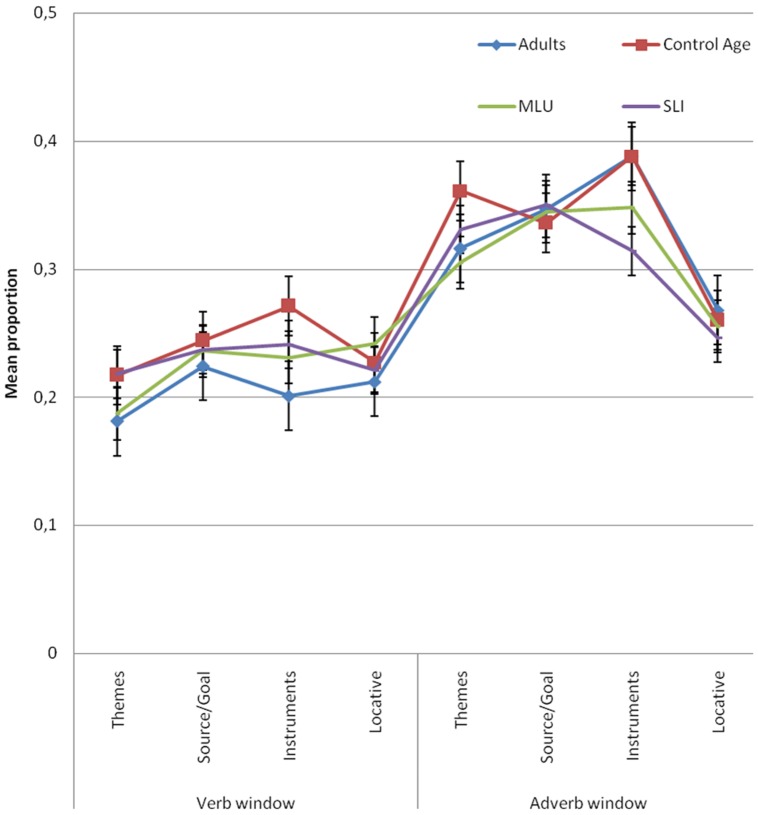
**Mean proportion of looks at theme (te), instrument (it), locative (la) and source/goal (lm) referents binned into the verb and adverb window by group**.

In the adverb window (2000–3000 ms), results showed significant differences by argument type [*F*_1_(3,306) = 10.821, *p* < 0.001; 𝜀^2^ = 0.096; *F*_2_(3,80) = 2.891, *p* = 0.04; 𝜀^2^ = 0.098] but not between groups [*F*_1_(3,102) = 1.044, *p* = 0.377; *F*_2_(3,80) = 0.725, *p* = 0.54]. The interaction between these two variables was not significant either [*F*_1_(9,306) = 0.802, *p* = 0.615; *F*_1_(9,80) = 0.222, *p* = 0.99]. *Post hoc* comparison revealed that the differences related to argument type were restricted to the comparison between locatives and the rest of the arguments. **Figure 6** shows differences of the mean of proportion of looks with regard to argument type in each group of the sample both for the verb and adverb windows.

## Discussion

The purpose of this study was to investigate if children with SLI use verbs to predict arguments and adjuncts. For this purpose, we used the visual world paradigm to compare anticipatory looks at the themes, sources/goals, instruments (arguments) and locatives (adjuncts). Results showed that the proportion of looks at the theme, source/goal and instrument referents was significantly higher than for looks at locatives. This pattern was found for adults and children with and without SLI.

This finding adds further support to previous claims that verbs implicitly introduce their arguments as the verb is recognized (e.g., Altmann and Kamide, 1999; Mauner and Koenig, 2000; Kamide et al., 2003a; Boland, 2005; Knoeferle et al., 2005; Knoeferle and Crocker, 2006, 2007). People use the meaning of the verb to anticipate upcoming information and to determine reference entities in the world during real-time comprehension. Altmann and Kamide (1999) found that the selectional information conveyed by a verb was used to anticipate upcoming themes. Later, Kamide et al. (2003a) found that verb-based information was used to anticipate goals. Knoeferle et al. (2005) found that verb-mediated visual event information allowed early real-time disambiguation in agent and patient role fillers. Boland (2005) found that recipients were introduced into the discourse by verb information, while locatives, benefactives and instruments were not. In the present study, we have found that themes, sources/goals and instruments were significantly more anticipated than locatives.

Unlike most of the previous studies, no differences were found in anticipatory looks at target images during the verb time window. This could probably be attributed to the flexible word order of Spanish, which allows the canonical positions of the verb’s arguments and adjuncts to be changed. For this reason, both arguments and adjuncts can be mentioned after the verb and compete in real time as the upcoming referent. This characteristic generates more uncertainty in speakers than when they are hearing the verb in languages with a stricter word order, such as English. However, similar slower time courses have been reported with adolescent English speakers (Brock et al., 2008). In this study, participants with and without autism spectrum disorder showed an increase of target fixations well before the onset of the target word (at around 480 ms post-verb).

Although these patterns are of interest, this work shows (contrary to our expectations) that children from an early age, including children with SLI, use selectional information conveyed by a verb to anticipate upcoming information in sentence comprehension. The account on verb semantic representation deficits predicted that children with SLI would be less accurate that age-matched controls in anticipating arguments and adjuncts. Moreover, they would show fewer differences in the anticipation arguments in comparison with adjuncts. However, these differences were not found in any of these aspects. Despite their linguistic deficits, children with SLI performed accurately in a real-time spoken language comprehension task that required linking perceived speech to a visual referent world. These children were able to use knowledge about the semantic requirements of a verb to compute likely referents for upcoming arguments and adjuncts before these constituents were even spoken aloud.

Therefore, the present results suggest that children with SLI do not suffer impairment in retrieving the verb’s semantic information in order to anticipate arguments and adjuncts in sentence comprehension: like adults and age-matched children, children with SLI can anticipate upcoming referents based on verb information.

Our results showed results similar to those of Andreu et al. (2013). They found that children with NL development, like children with SLI, use the lexically encoded implicit participant verb information to introduce forthcoming entities into their discourse. Similar results were found by Nation et al. (2003) with skilled and less-skilled child comprehenders, who did not differ in the speed of their anticipatory eye movements, suggesting normal sensitivity to linguistic constraints.

However, these results contrast with previous research on argument structure in language production. The children with SLI used significantly fewer argument structure types and verb alternations than age-matched children (Thordardottir and Weismer, 2002); they omitted more obligatory arguments compared to age-matched controls (Fletcher, 1991; Roberts et al., 1993; Grela and Leonard, 1997; Grela, 2003; Sanz-Torrent et al., 2011; Andreu et al., 2012) and committed errors with a much wider variety of verbs compared to MLU controls (King and Fletcher, 1993). In language production, verb semantics play a crucial role because they allow speakers to specify the number of arguments that accompany the verb, their thematic roles, their syntactic expression and the semantic restrictions their arguments have to comply with. For example, if a speaker wants to use a phrase containing the verb “to catch,” by activating its semantics he know that he must specify two arguments that will have the thematic roles of agent and theme and which will be specified by the following subcategorization frame: [NP] catches [NP]. In addition, both the agent and the theme must be animated entities in most situations. Good candidates to fulfill these functions are “fisherman” and “fish.” Therefore, the speaker can produce the following sentence: *The fisherman catches the fish*.

In contrast, in language comprehension, the semantics of the verb enables the anticipation of subsequent information, facilitating processing time. When a speaker listens to the phrase *the dog chases*... When the verb “chase” is processed, all the verb’s associated semantic information is activated. Verb semantics allow the speaker to know that the sentence requires a further argument, that this argument will have the thematic role of patient, that it will be syntactically expressed as a prepositional syntagma and that it must comply with various lexical-semantic restrictions, such as being a living being, being able to run away, etc. The speaker will even conclude that a good candidate to finish the sentence would be a “cat.”

It is difficult to explain why children with SLI have more problems using verb semantics in language production than in comprehension. Psycholinguistic literature have showed that language production and comprehension are separate processes (e.g., Bock, 1995; Levelt et al., 1999; MacDonald, 1999; Vigliocco and Hartsuiker, 2002; Thornton and MacDonald, 2003).

Moreover, some authors have argued that language comprehension precedes language production (e.g., Ingram, 1974; Bates et al., 1988) because previous results have found that children perform better on comprehension tasks that on production (e.g., Fraser et al., 1963). However, others have showed that the relationship between comprehension and production changes during language development (e.g., Bloom, 1974; Chapman and Miller, 1975). Data from children with SLI is also contradictory. For example, Hakansson and Hansson (2000) found that Swedish children with SLI scored higher on comprehension of relative clauses than on production at Time I but in reverse 6 months later (Time II).

We suggest that the different nature of the tasks may explain the differences in performance found in SLI between sentence production and comprehension. Firstly, production is a task of word retrieval whereas comprehension is a task of word recognition. Comprehension is faster, because listeners are able to recognize a referent even before the speaker has completed articulation (Tanenhaus et al., 1995). However, naming an object in a picture takes around 900ms for start word production (Snodgrass and Yuditsky, 1996). Moreover, in comprehension the processor must piece-together potentially ambiguous inputs, whereas the producer starts with a conceptual representation (Thornton and MacDonald, 2003).

In our study, children listened simple sentences while see a concrete visual context with four pictures depicted. In that case, they only had to activate the semantics of the verb and choose the best visual referent to finish the sentence. However, sentence production is more complex especially in spontaneous speech. In this situation children have to generate language himself creating a message from an idea that they want to communicate. This is the case, for example, of the study of Thordardottir and Weismer (2002), which analyzed speech samples from 50 children with SLI.

Future research should to explore verb semantics and their influence in sentence comprehension and production. Children with SLI exhibit problems in language comprehension but not in language production. Further studies should analyze the causes of these differences.

## Conflict of Interest Statement

The authors declare that the research was conducted in the absence of any commercial or financial relationships that could be construed as a potential conflict of interest.
