# Supplementary material for: Do Children with SLI Use Verbs to Predict Arguments and Adjuncts: Evidence from Eye Movements During Listening
Source: Front Psychol. 2016 Jan 6;6:1917. doi: 10.3389/fpsyg.2015.01917 (PMC4702442; doi:10.3389/fpsyg.2015.01917)
Supplement: Supplementary file 1 [file Data_Sheet_1.DOCX]

## **Appendix.**

The 24 sentences used in the study are:

**Themes**

La mujer compra cada día el pan en la panadería

(TARGET: pan; COMPETIDOR: panadería; DISTRACTORS: playa, sol)

*[The woman buys bread every day in the bakery*

*(TARGET: bread; COMPETITOR: bakery; DISTRACTORS: beach, sun)]*

El hombre lee con atención un cuento en la cama

(T: cuento; C: cama; D: armario, uvas)

*[The man carefully reads a story on the bed*

*(T: story book; C: bed, D: wardrobe, grapes)]*

La niña come despacio la tarta con la cuchara

(T: tarta, C: cuchara; D: sombrero, dinosaurio)

*[The girl slowly eats the cake with the spoon*

*(T: cake t, C: spoon; D: hat, dinosaur)]*

El niño lanza deprisa la pelota en la canasta

(T: pelota; C: canasta; D: palmera, nariz)

*[The boy quickly throws the ball in the basket*

*(T: ball; C: basket; D: palm, nose)]*

El niño recorta con cuidado una hoja con las tijeras

(T: hoja; C: tijeras; D: caramelo, martillo)

*[The boy carefully cuts a sheet with the scissors*

*(T: sheet; C: scissors; D: caramel, hammer)]*

La niña pinta con cuidado el dibujo con los colores

(T: dibujo; C: colores; D: cuchillo, sombrilla.

*[The girl carefully paints the drawing with the colors*

*(T: drawing; C: color; D: knife, umbrella)]*

**Sources and goals**

El niño va deprisa a la escuela con la mochila

(T: escuela; C: mochila; D: planeta, fuego)

*[The boy goes quickly to school with the backpack*

*(T: school; C: backpack; D: planet, fire*]

La niña viene con alegría de la feria con un muñeco

(T: feria; C: muñeco; D: sol, elefante)

*[The girl comes joyfully from the fair with a doll*

*(T: fair, C: doll, D: Sun, elephant)]*

El hombre cae de repente al pozo con el coche

(T: pozo; C: coche; D: camiseta, imán)

*[The man suddenly falls into the well with the car]*

*(T: well, C: car; D: shirt, magnet)*

El hombre entra despacio en casa con la maleta

(T: casa; C: maleta; D: luna, tractor)

*[The man comes slowly home with the suitcase*

*(T: house; C: suitcase; D: Moon, tractor)]*

La mujer se sienta con cuidado en el sofá con el cojín

(T: sofá C: cojín; D: cactus, león)

*[The woman sits carefully on the sofa with a cushion*

*(T: C sofa: cushion; D: cactus, lion)]*

La mujer sale con tristeza del hospital con muletas

(T: hospital; C: muletas; D: nevera, Tierra)

*[The woman sadly leaves the hospital with crutches*

*(T: hospital; C: crutches; D: fridge, Earth]*

**Locatives**

La niña camina cada día por el parque con los zapatos

(T: parque; C: zapatos; D: tobogán, televisión)

*[The girl walks every day through the park with the shoes*

*(T: park, C: shoes; D: slide, television]*

La mujer pasea despacio por la montaña con el bastón

(T: montaña, C: bastón; D: sol, lápiz)

*[The woman walks slowly through the mountain with her cane*

*(T: mountain, C: cane; D: Sun, pencil)]*

El hombre vuela deprisa por el cielo con el avión

(T: cielo, C: avión; D: casa, silla)

*[The man flies rapidly across the sky with the plane*

*(T: sky, C: plane; D: home, chair)]*

La niña duerme siempre en la cama con el osito

(T: cama; C: osito; D: árbol, bombilla)

*[The girl always sleeps in bed with the teddy bear*

*(T: bed; C: teddy bear; D: tree, bulb)]*

El niño canta con alegría en el escenario con la guitarra

(T: escenario, C: guitarra; D: Luna, olla)

*[The boy sings with joy on stage with the guitar*

*(T: stage C: guitar, D: Moon, pot)]*

El niño corre deprisa por el bosque con la radio

(T: bosque, C: radio; D: avión, ordenador)

*[The boy quickly runs through the forest with the radio*

*(T: forest, C: radio; D: aircraft, computer)]*

**Instruments**

El niño nada deprisa con el flotador en la piscina

(T: flotador; C: piscina; D: botas, castillo)

*[The boy swims quickly with the flotation ring in the pool*

*(T: float; C: pool; D: boots, castle)]*

La niña resbala de repente con el plátano en la acera

(T: plátano; C: acera; D: globo, parque)

*[The girl suddenly slips on the banana skin on the sidewalk*

*(T: banana; C: sidewalk; D: balloon, park)]*

El hombre navega despacio con el barco por el lago

(T: barco; C: lago; D: libro, camino)

*[The man slowly navigates with the boat on the lake*

*(T: boat; C: lake; D: book, road)]*

La mujer patina con cuidado con el patinete por las escaleras

(T: patinete; C: escaleras; reloj, jardín)

*[The women carefully skates down the stairs with the scooter*

*(T: scooter; C: stairs; D: clock, garden)]*

El hombre bucea despacio con el tubo en el mar

(T: tubo; C: mar; D: estrella, cama)

*[The man slowly dives into the sea with the snorkel*

*(T: tube; C: sea; D: star, bed)]*

La mujer esquía deprisa con el trineo por la montaña

(T: trineo; C: montaña; D: vaso, playa)

*[The Woman skis quickly down the mountain with the sled*

*(T: sled; C: mountain; D cup, beach)]*
